# Identification of genomic regions involved in tolerance to drought stress and drought stress induced leaf senescence in juvenile barley

**DOI:** 10.1186/s12870-015-0524-3

**Published:** 2015-05-22

**Authors:** Gwendolin G Wehner, Christiane C Balko, Matthias M Enders, Klaus K Humbeck, Frank F Ordon

**Affiliations:** Julius Kühn-Institut (JKI), Federal Research Centre for Cultivated Plants, Institute for Resistance Research and Stress Tolerance, Rudolf-Schick-Platz 3, Sanitz, 18190 Germany; Interdisciplinary Center for Crop Plant Research (IZN), Hoher Weg 8, Halle (Saale), 06120 Germany; Julius Kühn-Institut (JKI), Federal Research Centre for Cultivated Plants, Institute for Resistance Research and Stress Tolerance, Erwin-Baur-Str. 27, Quedlinburg, 06484 Germany; Martin-Luther-University Halle-Wittenberg, Institute of Biology, Weinbergweg 10, Halle (Saale), 06120 Germany

**Keywords:** Barley, Leaf senescence, Drought stress, GWAS, QTL

## Abstract

**Background:**

Premature leaf senescence induced by external stress conditions, e.g. drought stress, is a main factor for yield losses in barley. Research in drought stress tolerance has become more important as due to climate change the number of drought periods will increase and tolerance to drought stress has become a goal of high interest in barley breeding. Therefore, the aim is to identify quantitative trait loci (QTL) involved in drought stress induced leaf senescence and drought stress tolerance in early developmental stages of barley (*Hordeum vulgare* L.) by applying genome wide association studies (GWAS) on a set of 156 winter barley genotypes.

**Results:**

After a four weeks stress period (BBCH 33) leaf colour as an indicator of leaf senescence, electron transport rate at photosystem II, content of free proline, content of soluble sugars, osmolality and the aboveground biomass indicative for drought stress response were determined in the control and stress variant in greenhouse pot experiments. Significant phenotypic variation was observed for all traits analysed. Heritabilities ranged between 0.27 for osmolality and 0.61 for leaf colour in stress treatment and significant effects of genotype, treatment and genotype x treatment were estimated for most traits analysed. Based on these phenotypic data and 3,212 polymorphic single nucleotide polymorphisms (SNP) with a minor allele frequency >5 % derived from the Illumina 9 k iSelect SNP Chip, 181 QTL were detected for all traits analysed. Major QTLs for drought stress and leaf senescence were located on chromosome 5H and 2H. BlastX search for associated marker sequences revealed that respective SNPs are in some cases located in proteins related to drought stress or leaf senescence, e.g. nucleotide pyrophosphatase (AVP1) or serine/ threonin protein kinase (SAPK9).

**Conclusions:**

GWAS resulted in the identification of many QTLs involved in drought stress and leaf senescence of which two major QTLs for drought stress and leaf senescence were located on chromosome 5H and 2H. Results may be the basis to incorporate breeding for tolerance to drought stress or leaf senescence in barley breeding via marker based selection procedures.

**Electronic supplementary material:**

The online version of this article (doi:10.1186/s12870-015-0524-3) contains supplementary material, which is available to authorized users.

## Background

Barley (*Hordeum vulgare* L.) is one of the first cereals domesticated in the Fertile Crescent [1] and today it is the fourth most important crop species concerning acreage next to wheat, maize, and rice [2]. Worldwide, barley is mainly used for animal feed and malting and only a very small amount is used for direct human consumption and bakery. Average yield of barley on the worldwide level is 2.9 t/ha but in some European countries, e.g. Germany average yield is up to 6.5 t/ha [2]. Barley yield in many parts of the world is reduced by biotic stress but also by abiotic stress e.g. heat, salt, deficits in nitrogen nutrition and drought [3–6]. Especially, in the juvenile stages from sowing to tillering, drought can severely influence barley development already reducing the potential yield [7]. Research on drought stress tolerance has become more important worldwide as due to climate change the number of drought periods will increase in the future [8, 9]. Up to now, most studies conducted in barley focused on effects of terminal drought stress whereas drought in juvenile stages is less well documented [10].

Drought tolerance is a complex quantitative trait, that is controlled by various mechanisms [11, 12]. Abscisic acid (ABA) is a key phytohormone involved in adaption to environmental stresses and regulation of plant development. It promotes the closure of stomata under drought stress conditions initiated by a loss of turgor [13]. Furthermore, it increases the hydraulic conductivity of water, promotes chlorophyll breakdown and leads to leaf senescence [14]. Another relevant protein is ubiquitin which regulates the degradation of proteins [15, 16]. Moreover, late embryo abundant (LEA) proteins and heat shock proteins that are involved in the protection of functional proteins are induced in response to various abiotic stresses [17–19]. For example protein kinases and protein phosphatases which activate or deactivate proteins by phosphorylation and dephosphorilation [20], as well as the LEA protein dehydrin which is described to have different functions in different stresses [21] is often present along with drought stress [22]. A lot of parameters indicative for drought stress influenced by these and additional genes were analysed in different crops [23, 24]. For example, biomass production [25], yield [26], photosynthesis rate [27], as well as the content of free proline [28], total content of soluble sugars [29], or osmolality [30] are parameters which are affected by drought stress in barley.

Another factor relevant for yield improvement is leaf senescence [31], which is a natural degradation process at the final stage of the development of organs and plants. This process is divided into three steps and starts with reprogramming of gene expression to turn on senescence activating genes. Before programmed cell death in the terminal phase occurs, the second step in which nutrients and metabolites are transported from source (e.g. roots, leaves) to sink (e.g. fruits, seed) is important for yield and quality of the seeds harvested [32]. During this reorganization phase, degradation of chlorophyll and a decrease in photosynthesis is observed [33, 34]. Because of degradation of chlorophyll, yellowing of the leaves is a symptom of leaf senescence [35], which in many studies is rated visually [36, 37], but can be more precisely determined by Soil Plant Analysis Development (SPAD) readings which estimate leaf greenness [38]. Degradation of chlorophyll is regulated by chlorophyllase, pheophorbide α oxygenase and red chlorophyll catabolite reductase among others [34], but so far for regulation of leaf senescence only a few genes are known [39, 40]. Leaf senescence is a process which is influenced by a lot of external stress conditions e.g. drought stress [24, 41]. Stress often results in premature induction of leaf senescence and therefore leads to an inefficient recycling of resources and a massive yield loss [42–44]. In contrast, plants showing delayed leaf senescence under stress, represented by a “stay green effect”, minimize yield loss [45].

Genome wide association studies (GWAS) are a powerful tool to subdivide such complex pathways as drought stress and leaf senescence by the detection of quantitative trait loci (QTL) out of the regression analysis of genotypic and phenotypic data [19, 46–48]. Up to now, some QTLs involved in drought stress response were published in barley [49–52], whereas for leaf senescence only few QTLs are known [42, 53]. Besides this, QTLs which are involved in the response to other abiotic stresses, e.g. salt stress were identified in barley [6, 54]. Molecular markers, such as single nucleotide polymorphisms (SNP) flanking QTLs having a significant influence on the respective trait can be used for efficient marker assisted selection and smart breeding procedures [55].

The aim of the present study is therefore the identification of QTLs for drought stress and drought stress induced leaf senescence in early developmental stages of barley suited to be used in future barley breeding programs using GWAS followed by the identification of the function of these QTLs associated to respective traits.

## Material & methods

### Plant material and experimental setup

A set of 156 winter barley genotypes (Additional file 1) consisting of 113 German winter barley cultivars [49 two-rowed and 64 six-rowed, [56] and 43 accessions of the Spanish barley core collection (SBCC) [57] were used to investigate drought stress induced leaf senescence in juvenile barley plants. Drought stress was applied in greenhouses of the Julius Kühn-Institut in Groß Lüsewitz, Germany according to Honsdorf *et al.* [51]. Trials were conducted in a split plot design with three replications per genotype and variant (control, drought stress). Ten seeds of each accession were sown per plastic pot (16x16x16 cm) containing 1,500 g of a mixed clay soil ED73 (H. Nitsch & Sohn GmbH & Co. KG, Dorsten Germany). After germination, seedlings were reduced to seven plants per pot. Plants were grown under semi-controlled long day conditions in a temperature range from 20 to 22 °C at day (16 h) and 17 to 19 °C at night (8 h). If natural radiation was below 20 klx, additional light was applied from 6 a.m. to 10 p.m. Drought stress started at the primary leaf stage (BBCH 10) seven days after sowing (das). At this time watering of the stress variant was stopped till the soil reached 20 % of the maximal soil water capacity, and then this level was kept by weighting each pot and re-watering. Control plants were continuously watered to 70 % of the maximal soil water capacity. Water capacity was calculated of the saturated soil weight and drought weight according to DIN ISO 11465 1996–12 [58]. At the end of a four weeks stress period (BBCH 33) physiological traits were determined and above ground biomass was harvested (experimental setup A). Experimental setup A was repeated in three years. A modified experimental setup was conducted to optimize lightening conditions of the primary leaves for the measurement of leaf colour (SPAD) and electron transport rate at photosystem II (ETR) (experimental setup B). General settings were the same as in setup A, but only four plants were grown in smaller pots (12x12x12 cm) to allow wider spacing of pots and all leaves except the primary leaves were tied up to reduce shading. Experimental setup B was repeated in two years.

### Physiological parameters determined

Six physiological traits, i.e. leaf colour (SPAD), electron transport rate at photosystem II (ETR), content of free proline (CFP), content of soluble sugars (CSS), osmolality (OA) and the aboveground biomass yield (BY) were determined in the control and stress treatment. Measurement and sampling respectively were conducted on primary leaves.

Chlorophyll content which was used as the main indicator for drought stress induced leaf senescence was measured 33–34 das by Minolta SPAD readings (Konica Minolta Chlorophyll Meter SPAD-502 Plus, Osaka Japan), which gives a value for leaf colour. Three primary leaves of three plants for each pot were measured at five positions per leaf. These SPAD readings turned out to be correlated to the chlorophyll content analysed photometrically [59, 60]. Because of this relation, the chlorophyll content can be indirectly measured by SPAD [61].

At 34–35 das chlorophyll fluorescence was measured in all genotypes using light adapted plants with the OS1P-Chlorophyll Fluorometer (OPTI-SCIENCE, Hudson USA) in the middle of three primary leaves per pot at one position per leaf. The relative electron transport rate at photosystem II (PSII) (*ETR* = *Y*(*II*) * *PAR* * 0, 84 * 0, 5) was calculated including the photosynthetically active radiation (PAR), as well as the quantum photosynthetic yield of PSII (Y(II)) and constants representing light which is absorbed by the leaf (0.84) and light which is equally absorbed by PSI and PSII (0.5) [62].

At 36 das five primary leaves per pot were harvested and cut in pieces of 1 cm length for the analysis of CFP, CSS and OA. These samples were frozen in liquid nitrogen immediately and samples for CFP and CSS measurement were freeze dried. For CFP measurement the ninhydrin method [63] was applied, and for CSS measurement the anthron method [64] was used. Both traits were measured photometrical using a spectrophotometer. The concentration of these ingredients was determined with a standard curve calculated on a dry weight basis. To assess OA, frozen leaf samples were grinded in a swing mill (30/s for 3 min), filled up with 200 μl water and centrifuged at 15,000 rpm for 15 min to get cell sap for the measurement of osmolality with a freezing-point osmometer (Osmomat O-30 Gonotec, Berlin Germany). Osmolality was corrected for the water content of fresh and dry weight. Above ground biomass was harvested 36 das, too. Leaf material was dried in a compartment dryer at 105 °C and weighted.

For all traits an outlier test was calculated to exclude extreme deviations [65]. To get information on the stability of all analysed traits in response to drought stress compared to the control, the drought susceptibility index (DSI) was calculated [66] for each trait and across treatments according to the formula:

$$ \mathrm{D}\mathrm{S}\mathrm{I} = \left(1\ \hbox{--}\ \mathrm{LSMean}\ \mathrm{Trait}\ \mathrm{Genotype},\ \mathrm{S}\mathrm{tress}\ /\ \mathrm{LSMean}\ \mathrm{Trait}\ \mathrm{Genotype},\ \mathrm{Control}\right)\ /\ \mathrm{D},\  with $$

$$ D = 1\ \hbox{--}\ \left(\mathrm{LSMean}\ \mathrm{Trait}\ \mathrm{Assortment},\ \mathrm{Stress}\ /\ \mathrm{LSMean}\ \mathrm{Trait}\ \mathrm{Assortment},\ \mathrm{Control}\right) $$

DSI is a relative value estimated for each genotype and trait. According to the formula genotypes revealing a DSI close to one are highly susceptible to drought and those close to zero or showing a negative value are tolerant.

### Statistical analysis of phenotypic data

Statistical analyses were performed with SAS 9.3 [67]. Least square means (LSMeans) were calculated with GLM procedure for the replications of each genotype in the respective years and for both control and drought conditions. Descriptive statistics was calculated out of LSMeans by PROC UNIVARIATE. Analysis of variance (ANOVA) was calculated using PROC MIXED with genotypes (G), drought stress treatment (T) and GxT as fixed effects. Replication (R), year (Y) and row type are chosen as random factors. The heritability (h^2^) was calculated with SAS in two steps. First, the variance components for the genotypes (V_G_), variance associated with the genotype by year interaction (V_GY_) and V_E_ which is the error variance were calculated with PROC VARCOMP. Next, h^2^ was calculated with the following formula: *VG*/(*VG* + *V*GY/*Y* + *VE*/RY) for both well watered and drought stress conditions. Furthermore, the coefficient of correlation (PROC CORR) by Pearson was calculated with SAS based on LSMeans.

### Genotyping and genome wide association study (GWAS)

For genotyping the whole set of genotypes was analysed with the barley Illumina 9 k iSelect SNP-chip [68]. Population structure was calculated with STRUCTURE 2.3.4 [69] based on 51 simple sequence repeat (SSR) markers covering the whole genome. The STRUCTURE programme was run 20 times for pre-defined *k* (the number of population groups) from 1 to 5 each. To get the number of calculated subpopulations (k) with highest likelihood the procedure of Evanno *et al.* [70] was applied. An independent run with 500,000 iterations of a Monte Carlo Markov Chain with a length of the burn in period of 500,000 was conducted for the k with the highest likelihood to obtain the q-matrix. Kinship was calculated with SPAGeDi 1.3d [71] based on 51 SSRs and allele size correlation coefficient [72] with 5,000 permutations.

Out of 3,886 genetically mapped SNP markers (398 at 1H, 690 at 2H, 583 at 3H, 342 at 4H, 781 at 5H, 546 at 6H and 546 at 7H) [68], 3,212 polymorphic markers with minor allele frequencies higher than 5 % were taken into account. Based on these data and respective phenotypic data (LSMeans) GWAS was conducted applying a mixed linear model (MLM) using TASSEL 3.0 [73]. All results with p values <0.001 were considered as significant marker trait associations. Linkage disequilibrium (LD) was calculated on mapped polymorphic SNPs with R [74] by an estimate of the average decay [75] over all barley chromosomes.

Sequences of significantly associated SNP-markers (p <0.001) were downloaded from the James Hutton Institute [http://bioinf.hutton.ac.uk/iselect/app] and respective sequences were compared against the plant proteome in the UniProtKB/Swiss-Prot protein database by BlastX (Basic Local Alignment Search Tool, p <10^−5^ or query cover of minimum 80 % in NCBI [https://www.ncbi.nlm.nih.gov] accessed Oct 2014) to get information on the proteins coded by these sequences [76]. Using UniProt [77] the involvement of respective proteins in drought stress and leaf senescence processes was analysed. In a last step a genetic map with all significantly associated SNPs in genes coding for proteins known to be involved in drought stress tolerance was generated using MapChart 2.2 [78].

## Results

### Phenotyping

The experiments revealed variability for genotype and treatment in all analysed traits as shown in Table 1. For the traits biomass yield (BY), leaf colour (SPAD) and the electron transport rate (ETR) the mean values for the stress treatment were lower than in the well watered variant. An exception are some genotypes of the SBCC (SBCC 3, 12, 14, 76, 80, 138 and 140) showing no decrease in SPAD and ETR or even an increase, represented by negative values across treatments (DSI). In contrast to the above mentioned traits, osmolality (OA), content of free proline (CFP) and total content of soluble sugars (CSS) increased under drought stress.

**Table 1:** Descriptive statistics, heritability (h^2^) and number of significant (p <0.001) quantitative trait loci (QTL)

Trait^a^	Description	Treat.^b^	Unit	Min^c^	Max^c^	Mean^c^	SD^c^	CV^c^	LSD^c^	h^2^	No. QTL (SNPs)
BY	Total above ground biomass yield	Control	g	4.47	18.18	10.70	2.68	25.08	4.12	0.80	72 (179)
		Stress	g	2.73	9.98	5.57	1.83	32.80	1.45	0.58	19 (32)
		DSI	unit free	0.65	1.28	0.98					50 (87)
SPAD	Leaf colour	Control	unit free	4.70	48.80	35.76	8.33	23.30	23.39	0.64	8 (12)
		Stress	unit free	7.83	46.77	34.16	6.56	19.20	21.41	0.61	3 (6)
		DSI	unit free	−7.70	9.41	0.93					2 (2)
ETR	Relative electron transport rate at PSII	Control	Y(II) x PAR x 0,84 x 0,5	6.12	32.26	15.73	4.63	29.46	19.47	0.08	
		Stress	Y(II) x PAR x 0,84 x 0,5	2.00	30.70	13.41	4.54	33.85	16.38	0.50	2 (2)
		DSI	unit free	−4.09	4.82	0.90					
OA	Osmolality	Control	osmol kg^−1^	0.23	0.78	0.46	0.05	11.57	0.19	0.00	
		Stress	osmol kg^−1^	0.40	1.16	0.67	0.08	11.48	0.23	0.27	22 (29)
		DSI	unit free	0.44	2.00	1.02	0.26				1 (1)
CFP	Content of free proline	Control	μmol g^−1^	0.00	22.36	3.66	3.11	84.89	7.33	0.13	
		Stress	μmol g^−1^	1.02	78.57	23.94	16.86	70.43	44.30	0.29	
		DSI	unit free	0.00	3.46	1.15					1 (2)
CSS	Total content of soluble sugars	Control	μmol g^−1^	86.07	568.07	231.40	85.51	36.95	240.72	0.13	
		Stress	μmol g^−1^	164.61	981.40	419.64	164.03	39.09	411.52	0.30	1 (1)
		DSI	unit free	0.08	2.95	1.07					

^a^BY: biomass yield, SPAD: leaf colour, ETR: electron transport rate at PSII, OA: osmolality, CFP: content of free proline, CSS: total content of soluble sugars

^b^Control and drought stress treatment, as well as DSI: drought susceptibility index across treatments by Fischer & Maurer (1978)

^c^Minimum, maximum, mean, standard deviation in % (SD), coefficient of variation (CV) (standard deviation divided by mean) and least significant difference (LSD)

The coefficient of variation (CV) was comparable for control and drought stress treatment (Table 1) for all six traits. Heritabilities (h^2^) estimated ranged between 0 for OA to 0.80 for BY in the control treatment and 0.27 for OA and 0.61 for SPAD in the stress treatment. Generally, h^2^ was higher for the stress treatment except for BY and SPAD. Analysis of variance (ANOVA) revealed significant (p <0.001) genotype and treatment effects for all investigated traits and genotype x treatment interactions for BY, CFP and CSS (Table 2).

**Table 2:** Analysis of variance (ANOVA) of analysed traits showing F and p values

Trait^a^	Effect^b^	F value	P value
BY	Genotype	7.61	<.0001
	Treatment	10878.9	<.0001
	G x T	4.16	<.0001
SPAD	Genotype	8.81	<.0001
	Treatment	30.74	<.0001
	G x T	0.83	0.9348
ETR	Genotype	2.08	<.0001
	Treatment	43.69	<.0001
	G x T	0.97	0.6007
OA	Genotype	1.45	0.0004
	Treatment	3737.86	<.0001
	G x T	1.16	0.0962
CFP	Genotype	2.59	<.0001
	Treatment	2544.91	<.0001
	G x T	2.89	<.0001
CSS	Genotype	2.85	<.0001
	Treatment	1984.1	<.0001
	G x T	2.42	<.0001

^a^BY: biomass yield, SPAD: leaf colour, ETR: electron transport rate at PSII, OA: osmolality, CFP: content of free proline, CSS: total content of soluble sugars

^b^Genotype, Treatment and GxT: genotype x treatment interaction effect

To get information on the influence of the physiological parameters estimated on biomass yield as the indicator for drought stress and SPAD as the indicator for drought stress induced leaf senescence, correlations to these traits were calculated (Table 3). For control and stress treatment BY is significantly correlated to SPAD with r = 0.39 and r = 0.36, respectively. A significant correlation was also determined for CSS to SPAD with r = 0.42 and for CFP with r = 0.42 in the drought stress treatment whereas for the control treatment significantly negative correlations were found. Low but nevertheless significant correlations to SPAD were also detected for ETR and OA in the control treatment. Similar correlations were detected for BY. High and significant correlations were found between BY and CSS (r = 0.36) and CFP (r = 0.31) for the drought stress treatment. Under control conditions the SBCC being a sub-population of its own, influences the correlation by producing less BY which results in reduced shading of the primary leaves and a negative correlation especially to CFP and CSS. By correlating only the German cultivars, these effects are excluded and no correlations (r = −0.16 for CFP and r = −0.03 for CSS) were observed. ETR and OA were not significantly correlated to BY.

**Table 3:** Coefficient of correlation (PEARSON) for control and drought stress treatment

	Treatment	SPAD	ETR	OA	CFP	CSS
BY	Control	0.395***	0.091	−0.127	−0.328***	−0.220**
	Stress	0.361***	−0.087	−0.124	0.307***	0.367***
SPAD	Control		0.160*	−0.185*	−0.239**	−0.192*
	Stress		−0.105	0.034	0.425***	0.418***

^a^BY: biomass yield, SPAD: leaf colour, ETR: electron transport rate at PSII, OA: osmolality, CFP: content of free proline, CSS: total content of soluble sugars

r is significant with *p <0.05, **p <0.01 and ***p <0.001

### Genotyping

**Fig. 1:**
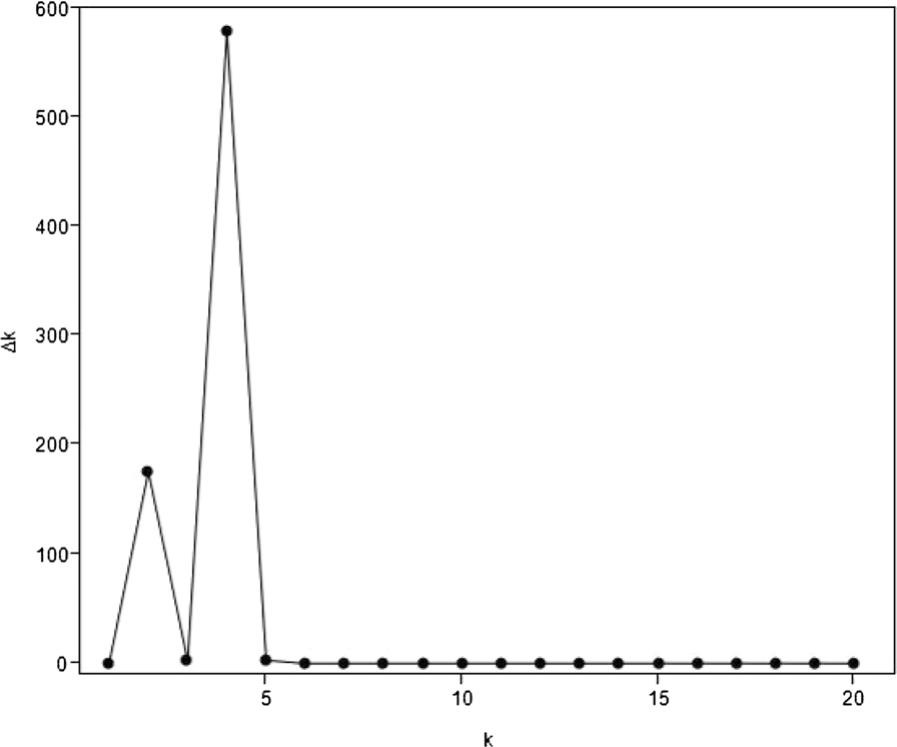
Optimal k of the population structure. The number of subpopulations within the set of barley genotypes was estimated at k = 4 by calculation described in Evanno *et al.* [70]

The set of genotypes was analysed with the 9 k iSelect SNP-chip available for barley. In summary 6,807 SNPs turned out to be polymorphic. Out of these, 3,212 SNPs are mapped on the seven barley chromosomes [68], showing a minor allele frequency (MAF) >5 %. This set of SNPs was used for the calculation of the linkage disequilibrium decay (LD), which turned out be on average 2.52 cM for this set of genotypes. The number of subpopulations was estimated at k = 4 (Fig. 1).

### Genome wide association study (GWAS)

**Fig. 2:**
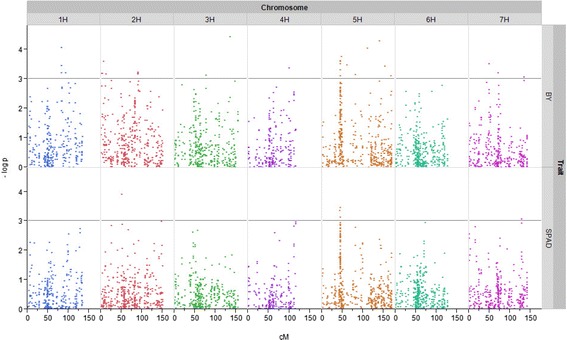
Manhattan plots. Showing –log p of association between Illumina SNPs and the analysed traits biomass yield (BY) and leaf colour (SPAD) against the position of the markers on all seven linkage groups (barley chromosomes) for stress treatment. Threshold indicates a significance level of p <0.001

Results of GWAS are shown in detail in Additional file 2 and summarized in Tables 4 and 5. 191 SNPs significantly (p <0.001) associated to traits estimated in the control variant, 70 significantly associated SNPs in the stress treatment and 92 significantly associated SNPs across treatments (DSI), were detected using the MLM analysis in TASSEL. Significant associations were found on all barley chromosomes. Most of the significant marker trait associations were located on barley chromosome 2H and 5H. A large number of SNPs on chromosome 5H around 45 cM turned out to be associated to SPAD and BY in the stress treatment (Fig. 2). Most significant associations for these traits were detected on chromosome 5H. The highest number of marker trait associations was detected for BY. No associations were observed for CFP in control and stress treatment, whereas across treatments one significant association was found. For ETR two significant associations and for CSS one significant association in the stress treatment explaining 5.5 % (ETR) and 1.6 % (CSS) of the phenotypic variance respectively were detected. The strongest association was observed on chromosome 1H for BY across treatments with a (−log p) value of 7.57 explaining 7.1 % of the phenotypic variance. For OA 29 significant associations were detected in the stress treatment located over all barley chromosomes, whereas in the control treatment no associations and across treatments only one association was found on chromosome 4H.

**Table 4:** Significant markers traits associations detected under drought stress conditions at a significance of p <0.001

Trait^a^	Number of genomic regions associated with the traits on the seven linkage groups (barley chromosomes)^b,c^							
	1H	2H	3H	4H	5H	6H	7H	Total QTL
**BY**	81.7 cM (3 SNP)	2 cM (3 SNP)	76.2 cM (1 SNP)	99.1 cM (1 SNP)	46.7 cM (8 SNP)		48.3 cM (1 SNP)	**19 (32 SNPs)**
	92.2 cM (1 SNP)	5.5 cM (1 SNP)	135.5 cM (1 SNP)		59.7 cM (1 SNP)		70.2 cM (1 SNP)	
		12.1 cM (1 SNP)			80.3 cM (1 SNP)		133.9 cM (1 SNP)	
		90.2 cM (3 SNP)			110.1 cM (1 SNP)			
					139.1 cM (1 SNP)			
					152.4 cM (1 SNP)			
					167.7 cM (1 SNP)			
**SPAD**		49.2 cM (1 SNP)			44.2 cM (4 SNP)		128.3 cM (1 SNP)	**3 (6 SNPs)**
**ETR**						59.4 cM (1 SNP)	2.1 cM (1 SNP)	**2 (2 SNPs)**
**OA**	116.8 cM (1 SNP)	51.8 cM (1 SNP)	2.4 cM (1 SNP)	52.3 cM (1 SNP)	46.5 cM (1 SNP)	10.3 cM (1 SNP)	106.5 cM (1 SNP)	**22 (29 SNPs)**
		60.8 cM (2 SNP)	36.8 cM (2 SNP)	110.2 cM (1 SNP)	55.7 cM (1 SNP)	47.5 cM (1 SNP)		
		81.5 cM (4 SNP)	51.8 cM (1 SNP)		95 cM (1 SNP)	51 cM (2 SNP)		
		135.8 cM (1 SNP)	61.9 cM (1 SNP)		137.9 cM (1 SNP)			
		146.5 cM (1 SNP)	89.4 cM (1 SNP)					
			100.7 cM (2 SNP)					
**CSS**	95.8 cM (1 SNP)							**1 (1 SNP)**
**Total QTL**	**4 (6 SNPs)**	**10 (18 SNPs)**	**8 (10 SNPs)**	**3 (3 SNPs)**	**12 (22 SNPs)**	**4 (5 SNPs)**	**6 (6 SNPs)**	**47 (70 SNPs)**

^a^BY: biomass yield, SPAD: leaf colour, ETR: electron transport rate at PSII, OA: osmolality, CSS: total content of soluble sugars

^b^One genomic region up to 2.6 cM (LD); the chromosomal position in cM was taken from the respective SNP with the highest R^2^

^c^Chromosome positions are based on Comadran *et al.* (2012)

**Table 5:** Significant blasted proteins related to drought stress or leaf senescence

Protein (Top BlastX hit with p <10^−5^ or query cover >80 %)	Protein abbr.	Accession	Function^a^	Marker^b^	Chr.^b^	Pos. in cM^b^	Trait^c^	Treat.^d^
Protease Do-like	DEGP2	[Swiss-Prot:O82261.2]	ls	BOPA1_8166-525	1H	47.5	BY	C, DSI
Cullin-1	CUL1	[Swiss-Prot:Q94AH6.1]	ls	SCRI_RS_85918	1H	47.7	BY	C
Serine/threonine-protein phosphatase PP1 isozyme 3	TOPP1	[Swiss-Prot:P48483.1]	ds	SCRI_RS_17924	1H	47.7	BY	C, DSI
Electron transfer flavoprotein-ubiquinone oxidoreductase	ETFQO	[Swiss-Prot:O22854.1]	ls	SCRI_RS_132604	1H	48.4	BY	C, DSI
ATP-dependent zinc metalloprotease	FTSH3	[Swiss-Prot:Q84WU8.1]	ls	BOPA1_2881-935	1H	81.7	BY	S
ABC transporter G family member 43	ABCG43	[Swiss-Prot:Q7PC81.1]	ds	BOPA2_12_31319	1H	92.4	SPAD	C, DSI
Probable pectinesterase 49	PME49	[Swiss-Prot:Q9LY18.1]	ds	SCRI_RS_235724	1H	95.8	CSS	S
Sucrose synthase 4	SUS4	[Swiss-Prot:Q9M111.1]	ls	SCRI_RS_239231	2H	49.2	SPAD	S
Metal-nicotianamine transporter YSL	YSL2	[Swiss-Prot:Q6H3Z6.2]	ds	SCRI_RS_221886	2H	80.9	BY	C
Glutamate dehydrogenase 2	GDH2	[Swiss-Prot:Q38946.1]	ls	BOPA1_3469-1152	2H	81.5	BY	C
Probable glutamate carboxypeptidase 2	AMP1	[Swiss-Prot:Q9M1S8.3]	ds	SCRI_RS_156090	2H	81.5	BY, OA	C, S
Probable phospholipid hydroperoxide glutathione peroxidase	GPX1	[Swiss-Prot:O23968.1]	ds	BOPA1_1635-691	2H	89.8	BY	S
Ethylene receptor 1	ETR1	[Swiss-Prot:Q9SSY6.1]	ls	SCRI_RS_185665	2H	114.9	BY	C, DSI
Cullin-3A 3B	CUL3A CUL3B	[Swiss-Prot:Q9ZVH4.1] [Swiss-Prot:Q9C9L0.1]	ls	BOPA1_3608-2133	2H	129.7	BY	C
Senescence-induced receptor-like serine/threonine-protein kinase	SIRK	[Swiss-Prot:O64483.1]	ls	SCRI_RS_8420	2H	139.9	BY	C
Putative F-box/LRR-repeat protein 21	FBL21	[Swiss-Prot:Q9M0U8.1]	ds	SCRI_RS_115423	3H	36.3	OA	S
1-aminocyclopropane-1-carboxylate oxidase	ACO1	[Swiss-Prot:Q9ZQZ1.1]	ds	SCRI_RS_167825	3H	100.3	BY, OA	C, S, DSI
Dehydrin	DHN 3	[Swiss-Prot:P12948.1]	ds	BOPA1_ABC13753-1-2-167	3H	105.3	BY	C
ABC transporter D family member 1	ABCA1	[Swiss-Prot:Q94FB9.1]	ds	SCRI_RS_142818	3H	148.2	BY	C
Abscisic acid receptor	PYL5	[Swiss-Prot:Q9FLB1.1]	ds	SCRI_RS_157396	4H	52.3	OA	S
Ethylene-responsive transcription factor	ERF011	[Swiss-Prot:Q9SNE1.1]	ls	SCRI_RS_9164	4H	113.7	BY	C, DSI
Nucleotide pyrophosphatase/phosphodiesterase	AVP1	[Swiss-Prot:Q687E1.2]	ds	BOPA1_9766-787	5H	44	BY	S
Abscisic acid-inducible protein kinase	TRIUR3	[Swiss-Prot:Q02066.1]	ds	SCRI_RS_102075	5H	44	SPAD	S
Serine/threonine-protein kinase	ATM	[Swiss-Prot:Q75H77.1]	ds	BOPA1_ABC08327-1-1-353	5H	44	SPAD	S
Serine/threonine-protein kinase	SAPK9	[Swiss-Prot:Q75V57.1]	ds	SCRI_RS_102075	5H	44	SPAD	S
Anthocyanin regulatory R-S protein	R-S	[Swiss-Prot:P13027.1]	ds	BOPA1_12045-83	5H	46.7	BY	S
Fasciclin-like arabinogalactan protein 2	FLA2	[Swiss-Prot:Q9SU13.1]	ds	BOPA1_5004-375	5H	83.5	BY	C
Serine/threonine-protein kinase	ATM	[Swiss-Prot:Q9M3G7.1]	ds	BOPA1_6315-914	5H	94.7	SPAD	C
Dehydration-responsive element-binding protein 1A	DREB1A	[Swiss-Prot:Q64MA1.1]	ds	BOPA2_12_30852	5H	95	BY, OA	C, S
Probable zinc metalloprotease EGY1	EGY1	[Swiss-Prot:Q852K0.3]	ls	SCRI_RS_208686	5H	137.4	BY	C, DSI
Cation/H(+) antiporter 2	CHX	[Swiss-Prot:Q9SAK8.1]	ds	SCRI_RS_160297	7H	2.1	ETR	S
Ethylene-responsive transcription factor	ERF062	[Swiss-Prot:Q9SVQ0.1]	ls	SCRI_RS_150783	7H	48.3	BY	S

^a^Function of the proteins related to drought stress (ds) or leaf senescence (ls), given by UniProt

^b^Markers and chromosome positions are based on Comadran *et al.* (2012)

^c^BY: biomass yield, CSS: total content of soluble sugars, ETR: electron transport rate at PSII, CFP: content of free proline, OA: osmolality, SPAD: leaf colour

^d^C: control treatment, S: stress treatment, DSI: drought susceptibility index across treatments by Fischer & Maurer (1978)

For SPAD as an indicator of leaf senescence, twelve significant (p <0.001) marker trait associations in the control treatment, six under stress conditions and two across treatments were detected. For the drought stress indicator biomass yield 179 significant marker trait associations were found in the control treatment, 32 in the stress treatment and 87 across treatments. Significant marker trait associations for BY were evenly distributed over all chromosomes. Out of these, eight were identical in the stress treatment, control treatment and across treatments and a high number of 65 marker trait associations were identical in control treatment and across treatments.

In total the 191 significant associations estimated in the control treatment account for 80 genomic regions (LD = 2.52 cM), the 70 significant associations determined in the stress treatment represent 47 genomic regions and the 92 significant associations across treatments (DSI) account for 54 genomic regions. By comparing the localisation of marker trait associations detected, it turned out that significant associations were found for different traits at same positions, e.g. on chromosome 2H at 50 cM for SPAD and OA in the stress treatment and at 120 cM for BY and SPAD in the control treatment. Furthermore, on chromosome 5H at 45 cM significant marker trait associations for BY, SPAD and OA were detected in the stress treatment, and at 95 cM significant associations for BY and SPAD in the control treatment.

Summarizing, overlapping of QTL was found across treatments and for different traits, especially for BY and SPAD, which are also significantly correlated (Table 3). One interesting QTL was observed on chromosome 5H at 45 cM where a significant association to BY and SPAD in the drought stress treatment was found, which is also within the LD of a significant association to OA (Table 4). Therefore, at this position a putative major QTL for drought stress and leaf senescence may be located.

**Fig. 3:**
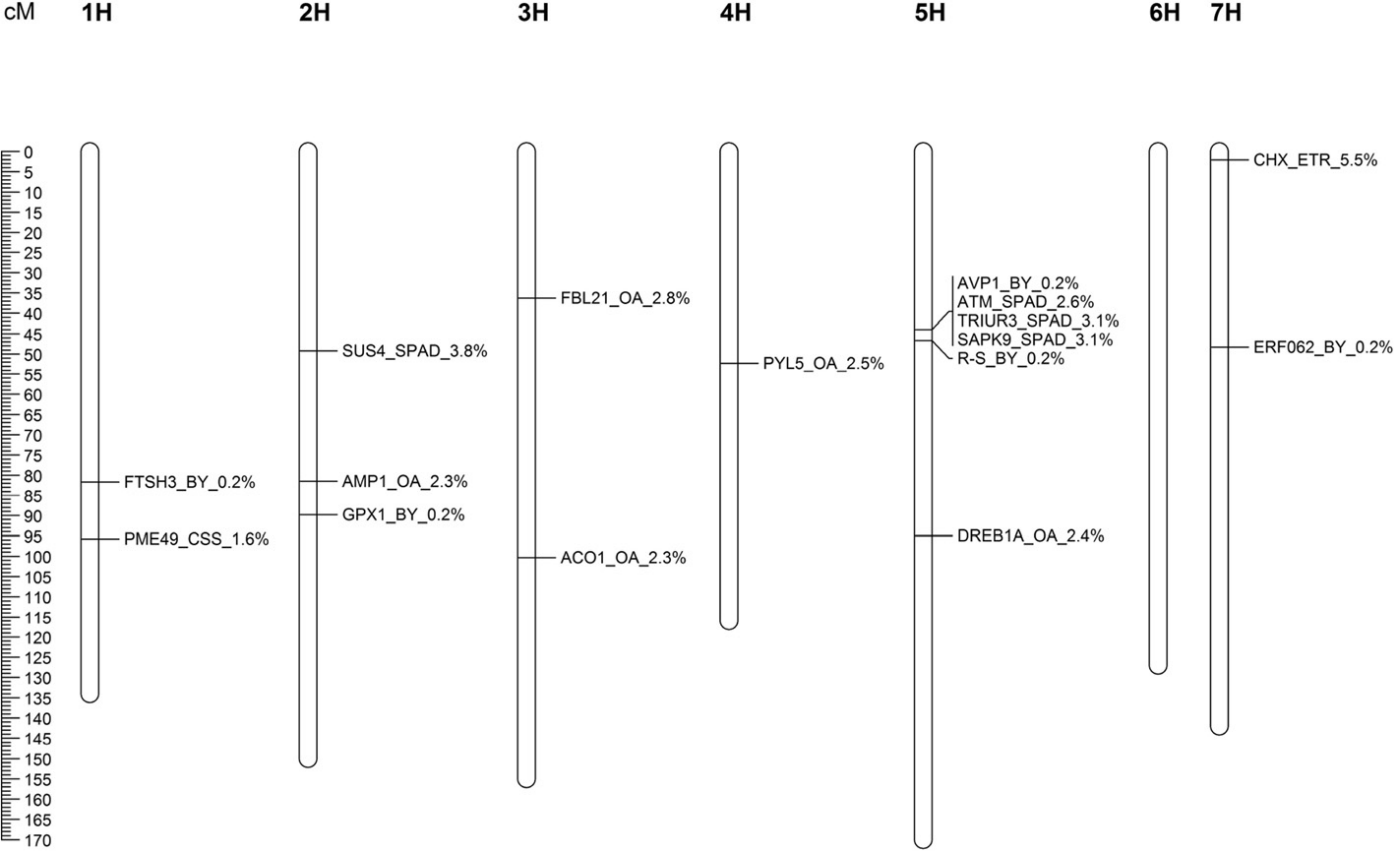
Genetic map. Shows QTL including the significant associated SNP marker positions for significant blasted proteins (BlastX) linked to drought stress or leaf senescence, related traits for drought stress treatment and percentage of phenotypic variance (explained R^2^ in %) of the SNPs for all linkage groups (barley chromosomes)

From the 353 significantly associated SNPs detected in the control treatment, stress treatment and across treatments (DSI), 127 proteins were identified by an NCBI Blast of the marker sequences. Out of these 19 proteins turned out to be related to drought stress, 10 proteins related to leaf senescence and 98 proteins turned out to be not related to drought stress or leaf senescence. Out of the 29 proteins for drought stress and leaf senescence (Table 5), 16 revealed associations under drought stress conditions. These were in a next step assigned to the barley chromosomes by the known genetic localization of respective SNPs (Fig. 3). Most of these were located at barley chromosome 2H and 5H, none were mapped on chromosome 6H.

## Discussion

Using the experimental design described above a clear cut influence of drought stress on biomass yield and physiological parameters was observed (Table 1). This effect of reduced biomass under drought conditions in juvenile stages was also reported in Honsdorf *et al.* [51] with an even higher reduction due to drought in early developmental stages of barley by Jamieson *et al.* [25]. In experiments on terminal drought stress application in barley this effect was not so pronounced [25, 79, 80] giving hint that barley is most susceptible to drought stress in early developmental stages. Furthermore, a reduction of the chlorophyll content under drought stress conditions in barley has been observed [80, 81], but there are also reports on adverse effects, e.g. for rapeseed or potato [82, 83], which may be due to a reduced leaf growth under drought stress conditions resulting in a reduced cell expansion leading to a relatively higher chlorophyll density in the leaves. In the present study measurement was done on primary leaves, which were fully expanded at the initiation of drought stress, so that this effect was excluded. For biomass yield a significant correlation to the leaf senescence parameter chlorophyll content was observed (Table 3). This correlation may be based on a true genetic relationship between these parameters, as it is also reported in drought stress field studies on wheat [59, 84] and in glasshouse experiments [85].

The electron transport rate at PS II as a parameter for the chlorophyll fluorescence decreased under drought stress indicating the degradation of chlorophyll during drought stress induced leaf senescence (Table 1), as already shown by Fang *et al.* [86], Li *et al.* [27], Netto *et al.* [87] and Silva *et al.* [88].

As expected, osmolality increased under drought stress thereby protecting cells against a turgor loss [30]. This is also reported in other drought stress studies on barley [29], but is more often detected in barley under salt stress conditions [89, 90]. For OA no correlation was found to BY, as also shown in a drought stress study on spring barley [91].

The amino acid proline has been described as an osmo-protectant [92] and is accumulated along with several abiotic stresses, such as drought stress, as seen in the present study (Table 1). This effect was also found in other drought stress studies, for example on barley in pot experiments [28], in greenhouse [93] and on *Arabidopsis thaliana* in climate chamber experiments [94]. The role of proline accumulation is still controversially discussed as it is described to function as a radical scavenger, antioxidant and is involved in the regulation of apoptosis and in seed development [95, 96, 97]. High correlations were detected for CFP to SPAD and BY giving hint that this trait is involved in drought stress tolerance and leaf senescence (Table 3). The correlation of the proline content to SPAD was also found in winter survival studies of barley [98] and in studies on salt stress tolerance in *Trigonella foenum-graecum* [99]. Up to now no correlations of CFP to BY under drought stress have been described for barley, but positive correlations to yield in wheat were observed under drought stress [100].

Soluble sugars are acting also as osmo-protectants and consequently like in our study an increase was detected in several drought stress experiments on barley [29], wheat [101], potato [102] and also pea [103]. Furthermore, studies showed that an increase of soluble sugars occurs along with leaf senescence [104] and that CSS was correlated to leaf senescence and biomass production [105, 106]. Interactions between sugar and ABA signalling may be responsible for the induction of senescence during drought stress [107].

Quite high values for the heritability of respective traits estimated under drought stress conditions (Table 1) give hint that such an experimental design is suited together with a set of diverse genotypes and the respective number of SNP-markers to detect QTLs using a genome wide association approach (GWAS). Like in other studies [49, 108, 109], the highest number of associations was detected for the traits with the highest heritability. In the present study these were SPAD and BY. Most associations were found on barley chromosomes 2H and 5H on which QTLs were located at 50 cM and at 45 cM, respectively. Also in other GWAS studies of barley significant QTLs for SPAD and BY were located on these chromosomes. Close to the QTL for SPAD located on chromosome 2H (50 cM) a QTL for SPAD under drought stress was also mapped by Li *et al.* [50]. Moreover, on chromosome 2H at 115 cM a QTL for SPAD was identified in a pot experiment with post-flowering drought stress [53]. QTLs for SPAD were also located on chromosome 2H at 102.7 cM and on chromosome 5H at 165.2 cM in Mediterranean dry land experiments (110), but no significant marker trait associations were detected at these positions in our experiments. The same holds true for a QTL for SPAD on chromosome 5H at 139 cM [49]. Varshney *et al.* [49] also detected a QTL for biomass yield on chromosome 5H at 95 cM and 156 cM, which is near to associations, which were found in our study on chromosome 5H at 152 cM and 167 cM. In addition, in the present study a QTL for SPAD and BY under drought stress treatment was detected on chromosome 5H at 45 cM, which has not been described before. Furthermore, a lot of significant marker trait associations were observed for osmolality under stress treatment distributed over all barley chromosomes. This was also reported for barley based on growth chamber drought experiments [29].

Proteins involved in drought stress and leaf senescence were detected by a blast of SNP marker sequences and it turned out that they are distributed over all barley chromosomes, except 6H with a focus on chromosomes 2H and 5H (Table 5). Most interesting proteins detected in the drought stress treatment (Fig. 3) are discussed in detail.

On chromosome 1H an ATP-dependent zinc metalloprotease (FTSH3) which is a regulator of heat shock proteins turned out to be associated to BY under drought stress. This protein is involved in the thylakoid formation and in the removal of damaged D1 in the photosystem II, preventing cell death under high-intensity light conditions. In interaction with heat shock proteins it reduces chlorophyll a/b ratios in heat tolerance regulation in *Arabidopsis thaliana* [111]. Heat stress often occurs simultaneously with drought and also leads to leaf senescence. Besides this, on chromosome 1H the pectin methylesterase 49 (PME49) was found by sequence alignment to be associated to CSS. This protein acts in the modification of cell walls via demethylesterification of pectin and turned out to be up-regulated by drought stress in rice [112]. It influences the mechanical stability of cell walls and thereby also of leaves.

On chromosome 2H a sucrose synthase 4 (SUS4) turned out to be associated with SPAD. This is a sucrose-cleaving enzyme that provides UDP-glucose and fructose for various metabolic pathways and is involved in nucleic acid break down during leaf senescence as revealed by expression analysis e.g. in cucumber and rapeseed [113]. The SNP marker with the homolog sequences to this protein was associated to the leaf senescence parameter SPAD at 49.2 cM. So there may be a direct relationship between the SPAD values and the activation of this enzyme, especially because this SNP marker explains 3.8 % of the phenotypic variance. Furthermore, a probable glutamate carboxypeptidase (AMP) revealed an association to OA which plays an important role in shoot apical meristem development and phytohormone homeostasis. By microarray analysis it turned out that AMP mediates ABA production and is involved in abiotic stress response such as drought stress in *Arabidopsis thaliana* [114]. Moreover, a marker with a sequence homologue to a phospholipid hydroperoxide glutathione peroxidase (GPX1) associated to BY was found which protects cells and enzymes from oxidative damage. Photometrical analyses of protein quantity and activity showed that the expression of GPX1 and GPX3 is reduced under drought stress and restored after recovery in winter wheat [115].

On chromosome 3H an F-box protein was detected (FBL21) associated to OA. These proteins are ubiquitin related and negatively regulate ABA mediated drought stress response in *Arabidopsis thaliana* [116]. Furthermore, an association of OA to 1-aminocyclopropane-1-carboxylate oxidase (ACO1) was detected which limits leaf growth by inhibiting the ethylene biosynthesis and so leads to drought tolerance. This was figured out in barley by expression analyses of protein related genes [117]. Surprisingly, in the control treatment associated to BY, a well known drought stress related protein, i.e. dehydrin (DHN) [118] was found which was also in another study located on chromosome 3H [119]. This protein belongs to the family of late embryogenesis abundant (LEA) proteins and is reported to be up-regulated in the protection mechanisms activated by plants in response to drought stress in wheat [120].

On chromosome 4H an ABA receptor (PYL5) was located and associated to OA that activates ABA signalling and ABA-mediated responses such as stomatal closure and germination inhibition. Immuno-detection experiments of protein extracts revealed that ABA signalling is involved in several stresses for example drought stress in *Arabidopsis thaliana* [121].

On chromosome 5H nucleotide pyrophosphatase/phosphodiesterase (AVP1) was found associated to BY which facilitates auxin transport by modulating apoplastic pH and regulating auxin-mediated developmental processes. Increased expression of protein related genes in transgenic barley confers tolerance to NaCl and to drought by increasing ion retention [122]. Furthermore, three protein kinases associated to SPAD were located on this chromosome regulating protein activity by phosphorylation. First a serine/threonine-protein kinase (ATM) which leads to stress induced programmed cell death, shown in *Arabidopsis thaliana* by expression profiles of protein related genes [123], second a serine/threonine-protein kinase (SAPK9) which is activated by hyperosmotic stress in rice [124] and third the abscisic acid-inducible protein kinase (TRIUR3) observed in wheat, which is also involved in dehydration stress response [125]. Moreover, an anthocyanin regulatory protein (R-S) was detected associated to BY. Anthocyanin is often accumulated in abiotic stress response, among others in drought stress with a photoprotective function as shown in *Arabidopsis thaliana* [94, 126]. All of these proteins showed homologies to sequences of SNPs at chromosome 5H around 45 cM and were associated significantly to BY or SPAD, representing an interesting candidate QTL for drought stress and leaf senescence. In addition, a dehydration-responsive element-binding protein (DREB1A) was found associated to OA, which delays water stress symptoms and promotes expression of drought tolerance genes in transgenic wheat [127].

On chromosome 7H another ethylene responsive protein was found to be associated to BY under drought stress. The transcription factor ERF062 is involved in the regulation of gene expression by stress factors (transcriptional repressors) and progression of leaf senescence in *Arabidopsis thaliana* [128]. Besides, on chromosome 7H the cation/H(+) antiporter 2 (CHX) was detected associated to ETR, which is important for pH gradients in the cell. This protein plays a vital role in maintaining both cellular and intercellular ionic balances under stresses such as drought stress as observed in *Arabidopsis thaliana* [129].

In summary the blast of the associated SNPs to protein data bases revealed many proteins which are known to be involved in drought stress response or leaf senescence, respectively giving hint that the GWAS approach is well suited for the genetic dissection of these traits in barley. Out of the QTL detected, the ones on chromosome 2H at 50 cM and chromosome 5H at 45 cM are of prime importance and may be involved in breeding barley for drought tolerance in the future due to the quite high amount of phenotypic variance explained.

## Conclusions

By GWAS marker trait associations for above ground biomass and physiological traits involved in drought stress tolerance and leaf senescence in early developmental stages of barley were detected. Major QTL for BY and SPAD under drought stress were located at chromosome 2H at 50 cM and chromosome 5H at 45 cM, giving hint that in these regions putative major QTLs for drought stress and leaf senescence are located. With respect to the QTL on chromosome 2H, QTLs for drought stress and leaf senescence were located at comparable positions in other GWAS studies while the one on chromosome 5H was detected for the first time. By BlastX of respective SNP carrying sequences, 29 proteins were identified being involved in drought stress or leaf senescence, respectively. Respective QTLs may be the starting point for marker based selection in barley for drought stress tolerance in the juvenile stage.

## Additional files

Additional file 1:
**Overview of the 156 analysed genotypes.** ^a^SBCC: Spanish Barley Core Collection. Additional file 2:
**Significant associations (p <0.001) of the genome wide association study and blasted proteins out of the marker sequences.** ^a^BY: biomass yield, CSS: total content of soluble sugars, ETR: electron transport rate at PSII, CFP: content of free proline, OA: osmolality, SPAD: leaf colour. ^b^C: control treatment, S: stress treatment, DSI: drought susceptibility index across treatments by Fischer & Maurer (1978). ^c^Markers and chromosome positions are based on Comadran et al. (2012).

## Abbreviations

e.g.: for example
i.e.: id est
GWAS: Genome wide association study
QTL: Quantitative trait locus
SNP: Single nucleotide polymorphism
ABA: Abscisic acid
LEA: Late embryogenesis abundant protein
SPAD: Soil Plant Analysis Development; measurement of chlorophyll content by colour
SBCC: Spanish Barley Core Collection
das: Days after sowing
ETR: Electron transport rate
CFP: Content of free proline
CSS: Total content of soluble sugars
OA: Osmolality
BY: Biomass yield
PSII: Photosystem two
PAR: Photosynthetic active radiation
DSI: Drought susceptibility index; value across treatments
LSMeans: Last Square Means
h^2^: Heritability
CV: Coefficient of variation
SSR: Simple sequence repeat
MAF: Minor allele frequency
k: number of subpopulations
MLM: Mixed linear model
LD: Linkage disequilibrium
Blast: Basic Local Alignment Search Tool
